# Anomalous Left Coronary Artery Originating From the Right Coronary Sinus With a Septal Curse in a Patient With Myocardial Infarction Due to Right Coronary Lesion: Innocent Until Proven Otherwise

**DOI:** 10.7759/cureus.74130

**Published:** 2024-11-21

**Authors:** Humberto Morais, Rafael Lacerda, Afonso Kapenda, Cláudio L Mbala, Telmo Martins, Elsa Fernandes, Mauer Gonçalves

**Affiliations:** 1 Cardiology Department, Hospital Militar Principal/Instituto Superior, Luanda, AGO; 2 Internal Medicine Department, Medical School of Juazeiro do Norte, Juazeiro do Norte, BRA; 3 Cardiology Department, Clínica Girassol, Luanda, AGO; 4 Cardiology Department, Multiperfil Clinic, Luanda, AGO; 5 Post-graduate Department, Multiperfil Clinic, Luanda, AGO; 6 Cardiology Department, Medical School, Agostinho Neto University, Luanda, AGO

**Keywords:** acute coronary syndrome, computed angiotomography, coronary angiography, coronary artery anomalies, coronary vessel anomalies

## Abstract

Coronary artery anomalies (CAAs) are rare and challenging, with increased diagnoses due to advanced cardiovascular imaging, even in low-income countries where diagnostic and therapeutic approaches can be difficult. This case report details a 65-year-old Black male patient with a history of hypertension and smoking who presented with a myocardial infarction. Despite no significant abnormalities apart from the infarction, invasive coronary angiography revealed a dominant right coronary artery (RCA) and an anomalous left main coronary artery (LMCA) originating from the right coronary sinus, bifurcating into the left anterior descending artery and circumflex artery. An 80% stenotic lesion in the distal RCA was treated with percutaneous transluminal coronary angioplasty (PTCA) and drug-eluting stent implantation. Coronary computed tomography angiography (CCTA) confirmed the findings, detailing the anomalous LMCA's course. This case underscores the rarity and clinical significance of CAAs, particularly an anomalous LMCA with a septal course, as a diagnostic challenge and the impact of course study on therapeutic decision-making. While conservative treatment is generally recommended, surgical intervention may be necessary for high-risk cases. For this patient, the current myocardial infarction was due to atherosclerotic disease in the RCA, effectively treated with PTCA and stent placement. The percutaneous treatment of RCA stenosis, despite the anomalous LMCA without significant atherosclerosis, appears to be effective and safe.

## Introduction

Coronary artery anomalies (CAAs) are rare but clinically significant, with an incidence between 0.3% and 5.6% [1,2]. A recent study in Angola showed a 0.72% incidence in patients undergoing coronary computed tomography angiography (CCTA) for suspected coronary disease [3].

In cases of the anomalous location of the coronary ostium in an improper sinus (right coronary artery (RCA) arising from the left anterior sinus or left coronary artery (LCA) arising from the right anterior sinus), the anomalous course may be (1) posterior atrioventricular or retrocardiac sulcus; (2) retroaortic; (3) between the aorta and the pulmonary artery (intramural or interarterial); (4) intraseptal; (5) anterior to the pulmonary outlet; and (6) posteroanterior interventricular groove [4]. The interarterial course is considered malignant. It is a common cause of sudden death in young people. It is postulated that exercise expands the aorta, which occludes the sharply angled slit-like orifice of the left main coronary artery. The remainder are considered benign [2,4]. There is no difference in approach solely due to the presence of a coronary anomaly, except when it presents with a clearly malignant course (interarterial). Other factors are important in defining the type of approach, even in the absence of anomalies, such as triarterial disease and the presence of a common trunk lesion. In such situations, coronary bypass is indicated.

Although CAAs are rarely reported in Angolan patients (due to the existence of few diagnostic centers located only in the capital, Luanda), highlighting the need for regional studies, this case exemplifies an incidental finding of a septal subtype that was not the direct cause of the patient's condition.

The aim of this study is to report a rare case of an anomalous left coronary artery originating from the right coronary sinus with a septal course, highlighting the diagnostic challenges and the importance of thorough evaluations for safe therapeutic decision-making in patients with concomitant coronary lesions. The study was conducted in accordance with CARE guidelines, calling attention to the importance of an interdisciplinary approach in managing complex cases involving the investigation of coronary anomalies and their correlation with the global burden of cardiovascular diseases.

## Case presentation

A 65-year-old Black man with poorly controlled hypertension and a history of smoking was admitted to a provincial hospital with chest pain and diagnosed with subacute myocardial infarction of the inferolateral wall. Initial treatment included antiplatelet therapy, simvastatin, and intravenous heparin. After stabilization, he was transferred to a tertiary hospital in Luanda for cardiac catheterization.

**Figure 1:**
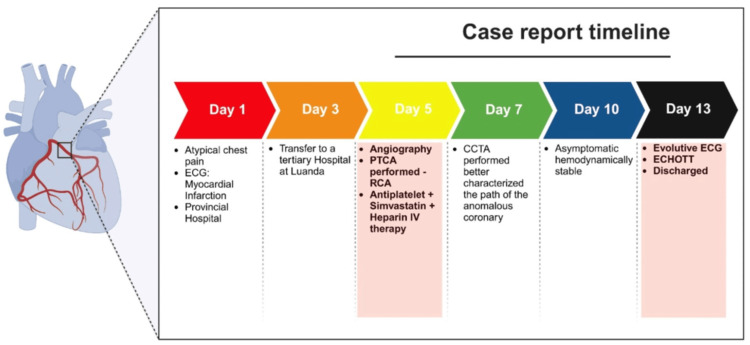
Case report timeline ECG: electrocardiogram, PTCA: percutaneous transluminal coronary angioplasty, RCA: right coronary artery, CCTA: coronary computed tomography angiography, ECHOTT: echocardiogram

Upon admission, the patient was asymptomatic with a pulse of 60 bpm and blood pressure of 106/60 mmHg. Laboratory results upon admission are shown in Table 1. An electrocardiogram (ECG) revealed sinus rhythm, Q waves in the inferior wall leads (II, III, and aVF), and negative symmetrical T waves in I, III, aVF, and V3-V6. Echocardiography showed a non-dilated left ventricle with preserved systolic function (left ventricular ejection fraction (LVEF): 65%) and meso-basal akinesia of the inferior wall. The timeline is shown in Figure 1.

**Table 1:** Laboratory tests LDL-c: low-density lipoprotein cholesterol, HDL-c: high-density lipoprotein cholesterol

Parameters	Patient values	Reference values
Hemoglobin	10.7 g/dL	13-16 g/dL
Glucose	134 mg/dL	70-110 mg/dL
Serum creatinine	1.33 mg/dL	0.72-1.25 mg/dL
Uric acid	10.48 mg/dL	3.5-7.2 mg/dL
Total cholesterol	132 mg/dL	<200 mg/dL
LDL-c	99 mg/dL	<100 mg/dL
HDL-c	54 mg/dL	>60 mg/dL
Triglycerides	101 mg/dL	<150 mg/dL

**Figure 2:**
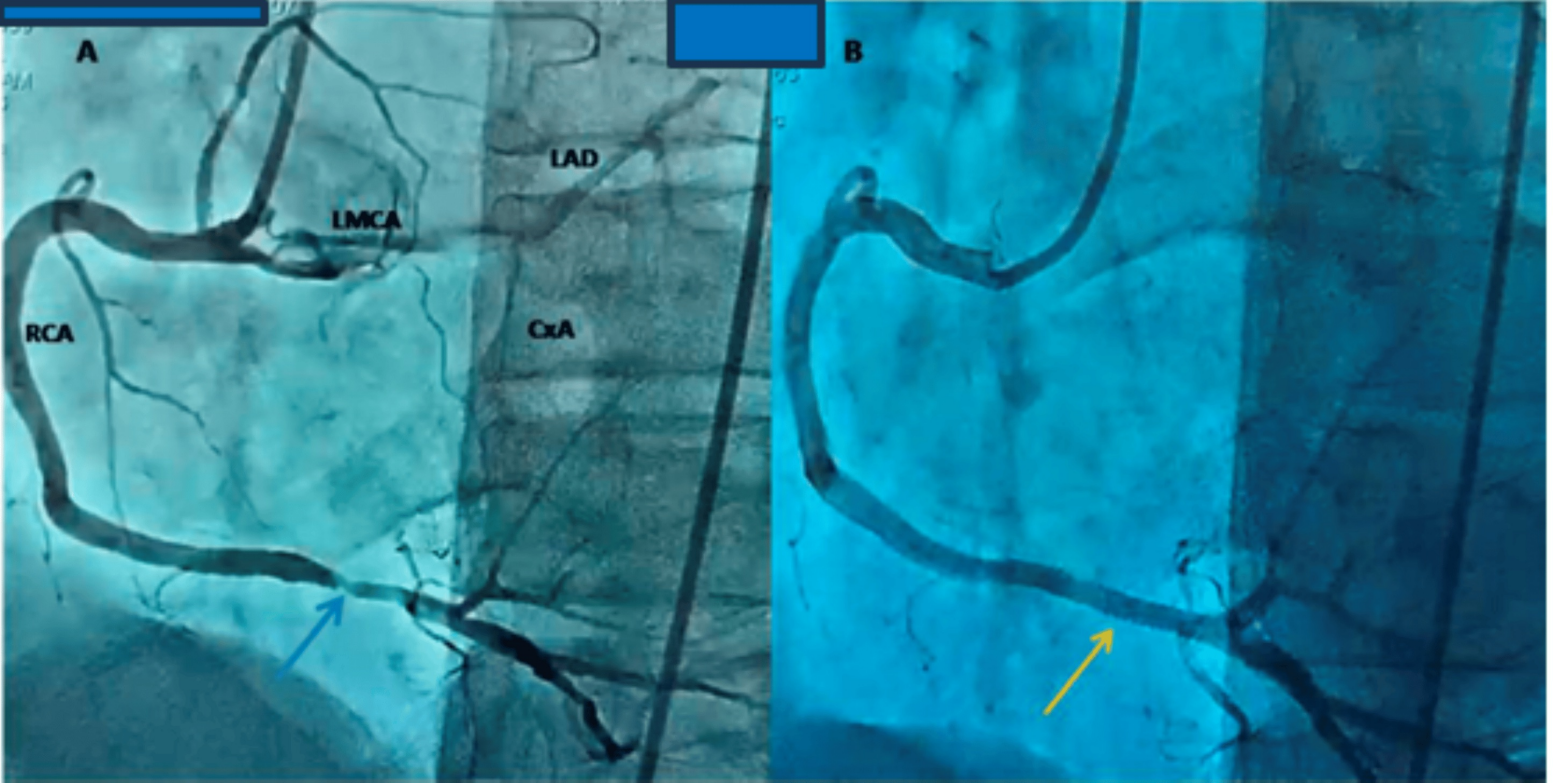
Invasive coronary angiography A: A single coronary artery with a dominant RCA with an 80% stenotic lesion (blue arrow) in the third distal segment. The left main coronary artery emerges from the RCA and originates the anterior descending artery, intermediate branch, and circumflex artery, without lesions. B: Status post drug-eluting stent placement (yellow arrow). CxA: circumflex artery, LAD: left anterior descending artery, LMCA: left main coronary artery, RCA: right coronary artery

Invasive coronary angiography revealed a single coronary ostium from the right coronary cusp, supplying the right coronary artery (RCA) and left main coronary artery (LMCA) (Figure 2A). The lesion in the distal RCA was treated with percutaneous stent implantation (Figure 2B).

**Figure 3:**
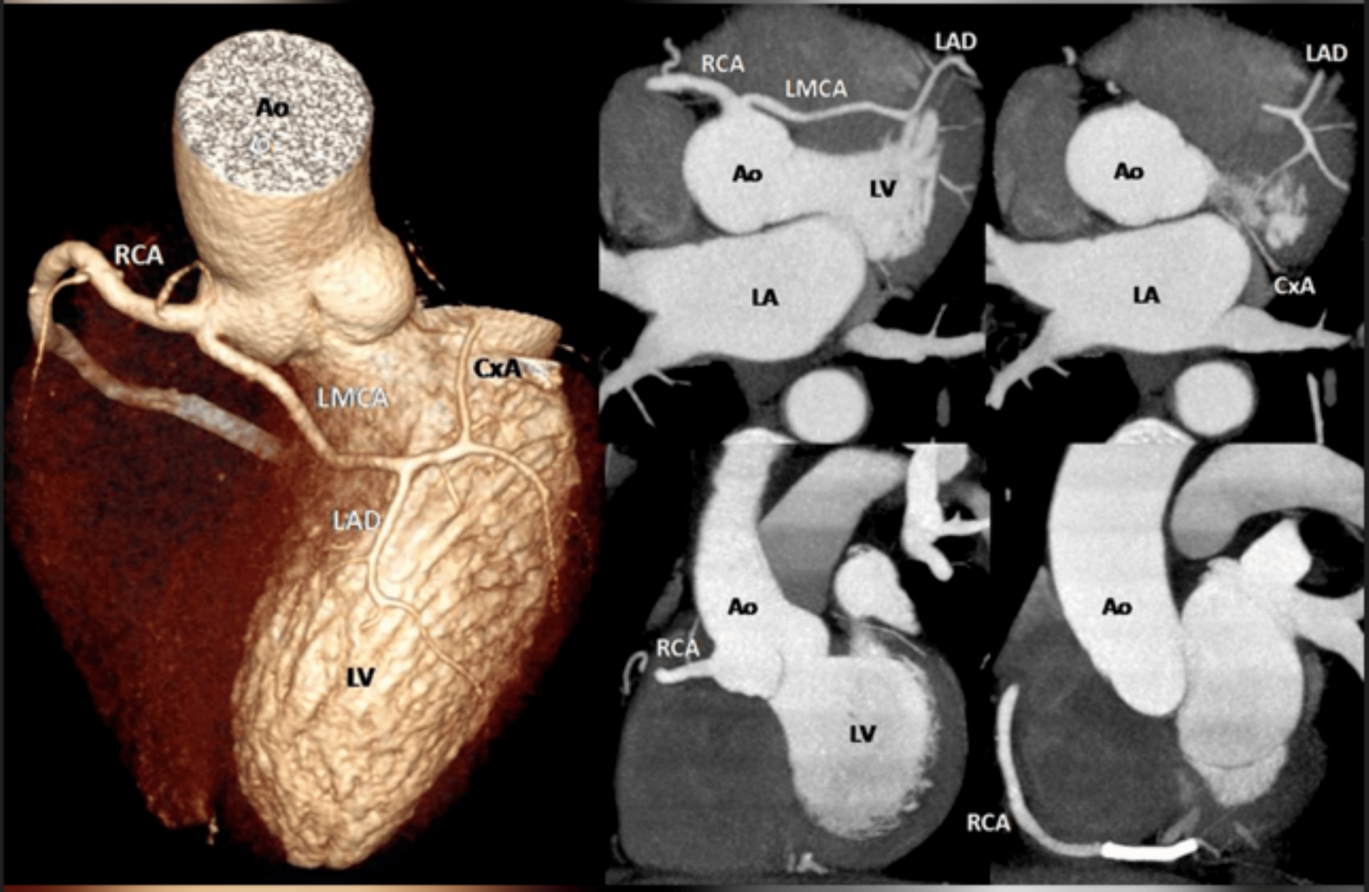
Coronary CT angiography Anomaly of the LMCA that emerges from the RCA with an intraseptal course directed to the middle one-third of the atrioventricular groove, where emerges the distal middle segment of the left anterior interventricular branch, a diagonal branch, and a thin ascending branch that corresponds to the circumflex artery. The stent placed in the distal middle third of the RCA is patent. Ao: aorta, CxA: circumflex artery, LA: left atrium, LAD: left anterior descending artery, LMCA: Left main coronary artery, LV: left ventricle, RCA: right coronary artery

CCTA confirmed the anomalous aortic origin of the coronary artery, showing a dominant RCA with a patent stent, and an anomalous LMCA from the right common coronary sinus with an intraseptal course (Figure 3). The patient was discharged and referred to an outpatient clinic, currently asymptomatic and on medication including dual antiplatelet therapy (aspirin and clopidogrel), statins (atorvastatin), amlodipine (calcium channel blocker for blood pressure control), and bisoprolol (beta-blocker aimed at reducing myocardial demand through heart rate reduction). The patient was referred for consultation one month after discharge, and a quarterly follow-up was scheduled for the first year.

## Discussion

The global incidence of CAAs is between 0.3% and 5.6%, with the anomalous origin of the LMCA from the right coronary sinus being very rare (up to 0.017% of coronary angiographies and 1.3% of all coronary anomalies) [1,2].

Most patients with CAAs are asymptomatic. The diagnosis is often incidental during coronary angiography or CCTA performed for suspected coronary artery disease, as in the present case [5-13]. The clinical significance depends on the origin and course of the anomalous artery, the perfused myocardium area, and the presence of atherosclerotic coronary disease [3]. Symptoms of myocardial ischemia can result from the coronary anomaly or concomitant atherosclerotic disease, complicating the differentiation of ischemic symptom origins only in light of the clinical context. Thus, especially in the case of malignant or clinically ambiguous courses, according to the literature, ischemia-inducing tests gain importance [11].

Defining the course of the anomalous coronary artery (ACA) is crucial as it affects clinical presentation and treatment. The interarterial course is malignant, potentially causing angina refractory to medical treatment, ventricular tachycardia, or sudden death, especially in young patients [14], constituting an indication for surgical treatment [3]. In addition, a triarterial disease or involvement of a common trunk would also be an indication for surgical intervention. Since none of the previous situations were observed in the present case, percutaneous transluminal coronary angioplasty (PTCA) was the reperfusion technique chosen. The anterior and posterior courses are benign, while the malignancy of the intraseptal course, as in the case discussed, is considered malignant by some because it can cause disabling angina and myocardial ischemia [14] and others benign [15].

Percutaneous treatment of significant atherosclerotic coronary disease of the RCA in the presence of an anomaly of the LMCA originating from the contralateral coronary sinus, with a malignant or undefined course, poses a great challenge. There is no consensus on the ideal approach to the diagnosis and treatment of these patients, due to the scarcity of cases reported in the literature.

We postulate that, to perform it safely, it is desirable to ensure the absence of ischemia in the territory of the anomalous coronary artery (ACA) before the percutaneous procedure using an ischemia induction test such as myocardial perfusion scintigraphy, stress echocardiography, or conventional exercise testing. If ischemia-inducing tests are not performed in the territory of the anomalous coronary artery (ACA), the sole approach to the right coronary artery (RCA) may underestimate and overlook potential ischemic injury in the left coronary territory. This could lead to the progression of myocardial damage and a worse clinical prognosis for the patient, potentially resulting in heart failure or refractory angina.

In the present case, CCTA performed after percutaneous treatment of the RCA lesion showed that the course of the anomalous artery was intraseptal. Furthermore, no ischemia-inducing tests were performed before percutaneous treatment of the RCA lesion. The team decided to treat the RCA lesion because it was considered the culprit lesion in the acute event. At the same time, they found no atherosclerotic lesions in the anomalous coronary artery, and the procedure was considered safe.

This case raises the question of what is the best approach to treating the right coronary artery lesion that is responsible for the coronary event in the presence of a left coronary artery anomaly with a malignant course. Should we always first investigate whether there is ischemia in the territory of the anomalous coronary artery (ACA)?

To answer this question, we reviewed PubMed using the MeSH descriptors "coronary sinus", "sinus of Valsalva", and "anomalous left coronary artery" over the last 30 years and found nine patients similar to ours (Table 2).

**Table 2:** Case reports over the last 30 years about patients with obstructive lesion in the RCA and anomalous LCA with a malignant or unknown course without significant atherosclerosis (search through PubMed/NCBI in July 2024) AMI: acute myocardial infarction, CABG: coronary artery bypass grafting, CT: computed tomography, IVS: interventricular septum, LAD: left anterior descending artery, LCA: left coronary artery, LCS: left coronary sinus, LCX: left circumflex artery, LMCA: left main coronary artery, PCI: percutaneous coronary intervention, RCA: right coronary artery, RCS: right coronary sinus, RCA: right coronary artery, SCA: single coronary artery, SPECT: single-photon emission computed tomography, TIMI: thrombolysis in myocardial infarction, T2DM: non-insulin-dependent type II diabetes mellitus

Index patient	Comorbidities	Clinical presentation	Diagnostic method and description of anomaly	Interventional therapy	Clinical outcome	Author and year
63 years old, male, ethnicity not specified	Active smoking, hypertension	Stable angina lasting for months	Coronary angiography showed that LMCA and RCA originated from the RCS. There was a 99% stenotic lesion in the RCA.	The intervention in the RCA involved balloon angioplasty and placement of three drug-eluting stents.	Two days later, the patient was discharged.	Gishto et al. (2024) [5]
63 years old, male, ethnicity not specified	Hypertension, T2DM with polyneuropathy and retinopathy, overweight	Progressive dyspnea with clinical signs of heart failure	Coronary angiography revealed the origin of the LMCA from the RCS. The RCA was occluded in its posterior ventricular branch. Coronary angiotomography showed a common trunk originating from the RCS with an intramyocardial course of the LCA.	Percutaneous transluminal angioplasty with implantation of a drug-eluting stent at the target lesion.	No evidence of ischemia on SPECT imaging control at one and six months	Mrad et al. (2023) [6]
68 years old, male, ethnicity not specified	Hypertension, diabetes, cocaine use	Angina pectoris for three weeks with acute worsening upon cocaine use	Coronary angiography showed 50% stenosis in the middle segment and 100% stenosis in the distal portion of the RCA. The LMCA arises from the RCS and shares a single ostium with the RCA.	The patient had a residual 100% stenosis with TIMI grade 0 flow and opted for medical treatment. He underwent CABG at another institution.	The patient was briefly readmitted to the prior center due to deconditioning after CABG.	Shtembari et al. (2023) [7]
68 years old, male, ethnicity not specified	No reported comorbidities	Angina (no mention of exertional correlation) lasting for two days	Coronary angiography showed a common origin of both RMCA and LMCA from the RCS, with obstructive disease in the middle segment of the RCA.	The patient was treated clinically and discharged with antiplatelets, beta-blockers, and nitrates.	The patient was progressing well.	Hirachan et al. (2017) [8]
56 years old, female, ethnicity not specified	No reported comorbidities	AMI of the inferior wall, Killip class I, treated with thrombolysis	Coronary angiography showed SCA originating from the RCS. Computed tomography confirmed the interarterial course of the LMCA. Nuclear stress scintigraphy showed no stress-induced ischemia in the LAD.	Successful PCI was performed using drug-eluting stents in the proximal and middle lesions of the RCA.	The patient was progressing well.	Thummar et al. (2014) [9]
68 years old, male, ethnicity not specified	Hypertension, T2DM	Recurrent atypical chest pain	Coronary angiography showed that the LMCA originated near the RCA and bifurcated into the LAD and LACX, with a 70% lesion in the mid segment of the RCA.	Percutaneous coronary intervention and stent placement were performed.	The author did not report follow-up after intervention.	Cakar et al. (2010) [10]
61 years old, male, ethnicity not specified	Previous myocardial infarction, active smoking	Prolonged angina associated with intense emotional stress	Coronary angiography showed the anomalous origin of the LMCA from the RCS and an RCA with significant stenosis. CT angiography showed that the anomalous LCA artery exhibited an interarterial course. Stress echo revealed hypokinesia in the mid and apical inferior segments, along with persistent akinesia in the basal posterior segment, associated with angina symptoms.	Revascularization of each of the right coronary artery lesions by angioplasty and stent placement were performed.	The patient was progressing well.	Balaguer-Malfagón et al. (2005) [11]
50 years old, male, ethnicity not specified	Active smoking	Typical angina pectoris of recent onset	Coronary angiography showed a SCA that originated from the RCS. The course of the LCA was interarterial. A severe stenosis in the RCA was also demonstrated.	Despite optimal medical treatment, the patient was admitted two weeks later for PCI of the RCA due to angina.	He was discharged two days after the procedure without symptoms.	Altun et al. (2003) [12]
54 years old, male, ethnicity not specified	No reported comorbidities	Unstable angina pectoris	Coronary angiography showed that both the RCA and LCA arose separately from the RCS. The RCA showed an 80% stenosis. LMCA took an intramyocardial course. Exercise treadmill thallium scintigraphy showed an absence of ischemia in the territory supplied by the LCA.	Percutaneous coronary angioplasty of the RCA stenosis with stent implantation was performed.	Five months later, the patient underwent a repeat exercise treadmill test with thallium scintigraphy, which showed normal myocardial perfusion.	Rentoukas et al. (2002) [13]

The concern to evaluate the existence of ischemia in the territory of the anomalous coronary artery prior to percutaneous treatment of the RCA lesion was assessed in only three patients using perfusion scintigraphy in two patients [9,13] and stress echocardiography in one [11]. Of these three patients, only one patient reported by Balaguer-Malfagón et al. [11] had ischemia observed in the territory of the ACA.

However, as in our case, in four patients, percutaneous treatment of the RCA lesion was performed solely based on the absence of atherosclerotic disease in the anomalous coronary artery, without prior ischemia-inducing testing. Despite this, no complications were recorded during the procedure [5,6,10,12].

## Conclusions

This case illustrated the important issue of the need to study ischemia in the territory of the ACA with a malignant course and free of significant atherosclerosis in initial angiography. As noted in the literature, an ischemic process was identified in one of the three cases where ischemia was studied in the territory of the ACA. As this analysis was conducted based on a small sample (due to the scarcity of similar reported cases), further studies are necessary to establish the safety of an approach to the RCA lesion without ischemic-inducing tests in contralateral septal coronary anomalies. Advanced imaging techniques, such as coronary computed tomography, stress echocardiography, and nuclear stress scintigraphy are essential for characterizing CAA and assisting in treatment planning.
